# Antibacterial Fusion Proteins Enhance *Moraxella catarrhalis* Killing

**DOI:** 10.3389/fimmu.2020.02122

**Published:** 2020-09-02

**Authors:** Maisem Laabei, Lucie Colineau, Serena Bettoni, Karolina Maziarz, David Ermert, Kristian Riesbeck, Sanjay Ram, Anna M. Blom

**Affiliations:** ^1^Division of Medical Protein Chemistry, Department of Translational Medicine, Faculty of Medicine, Lund University, Malmö, Sweden; ^2^Department of Biology and Biochemistry, University of Bath, Bath, United Kingdom; ^3^Clinical Microbiology, Department of Translational Medicine, Faculty of Medicine, Lund University, Malmö, Sweden; ^4^Division of Infectious Diseases and Immunology, University of Massachusetts Medical School, Worcester, MA, United States

**Keywords:** pathogen, *Moraxella catarrhalis*, fusion proteins, complement, antibacterial

## Abstract

*Moraxella catarrhalis* is a human-specific commensal of the respiratory tract and an opportunistic pathogen. It is one of the leading cause of otitis media in children and of acute exacerbations in patients with chronic obstructive pulmonary disease, resulting in significant morbidity and economic burden. Vaccines and new immunotherapeutic strategies to treat this emerging pathogen are needed. Complement is a key component of innate immunity that mediates the detection, response, and subsequent elimination of invading pathogens. Many pathogens including *M. catarrhalis* have evolved complement evasion mechanisms, which include the binding of human complement inhibitors such as C4b-binding protein (C4BP) and Factor H (FH). Inhibiting C4BP and FH acquisition by *M. catarrhalis* may provide a novel therapeutic avenue to treat infections. To achieve this, we created two chimeric proteins that combined the Moraxella-binding domains of C4BP and FH fused to human immunoglobulin Fcs: C4BP domains 1 and 2 and FH domains 6 and 7 fused to IgM and IgG Fc, respectively. As expected, FH6-7/IgG displaced FH from the bacterial surface while simultaneously activating complement via Fc-C1q interactions, together increasing pathogen elimination. C4BP1-2/IgM also increased serum killing of the bacteria through enhanced complement deposition, but did not displace C4BP from the surface of *M. catarrhalis*. These Fc fusion proteins could act as anti-infective immunotherapies. Many microbes bind the complement inhibitors C4BP and FH through the same domains as *M. catarrhalis*, therefore these Fc fusion proteins may be promising candidates as adjunctive therapy against many different drug-resistant pathogens.

## Introduction

*Moraxella catarrhalis* is a Gram-negative diplococcus which commonly colonizes the nasopharyngeal cavity of humans asymptomatically (1). However, in recent years, a greater appreciation of the virulent nature of this bacterium has emerged and *M. catarrhalis* is now considered an opportunistic pathogen (1, 2). *M. catarrhalis* is the third most common cause of acute sinusitis and otitis media in children and is responsible for a variety of lower respiratory tract infections in immunocompetent hosts and patients with chronic lung disease (2, 3). Rarely, *M. catarrhalis* can cause bacteraemia and pneumonia in immunocompromised individuals (4).

Humans have evolved a variety of mechanisms to evade infections caused by a multitude of microbial pathogens. The complement system represents an effective arm of innate immunity involved in detecting, labeling and eradicating potential microbial threats. Bacterial activation of complement is mediated by specific recognition molecules, which bind to conserved structures on the bacterial surface, initiating an enzymatic cascade resulting in the formation of the C3 convertases and cleavage of C3, the central protein of the complement cascade (5, 6). A strong activator of the classical pathway is the recognition subunit of the C1 complex, C1q (7). C1q interacts with high avidity with the Fc region of clustered immunoglobulin (Ig)Gs or multivalent IgM molecules and in conjunction with serine proteases, C1r and C1s, initiates proteolytic events resulting in C3 convertase formation (7). C3 can be cleaved into multiple fragments with opsonic properties and when deposited on the microbial surface, can interact with complement receptors expressed on the surface of professional phagocytes culminating in uptake and destruction of the pathogen (8). Further activation and processing of C3 forms C5 convertases, that cleave C5 into C5a, a potent chemoattractant, and C5b, an essential building block of the membrane attack complex (MAC). Interaction of C5b with complement proteins C6 through C9 results in formation and insertion of MAC leading to a reduction in membrane potential and bacterial lysis of Gram-negative bacteria (5, 6).

To prevent complement destruction of host cells, a suite of soluble and cell surface regulators maintain complement homeostasis (9). Two soluble proteins, Factor H (FH) and C4b-binding protein (C4BP) are pivotal for preventing unwanted complement activation, both exerting their influence at the level of C3 convertase inhibition (9–11). FH is the major soluble inhibitor of the alternative pathway (AP), binds to C3b via complement control protein (CCP) domains 1–4 and accelerates the decay of the alternative C3 convertase while also acting as a cofactor for the serine protease, factor I mediated inactivation of C3b (9, 10). C4BP is the major inhibitor of the classical and lectin pathways, interacting with and limiting the function of complement protein C4b (9, 11). In similar fashion to FH, C4BP acts as a cofactor for both FI proteolysis of cell-bound and soluble C4b, disrupting formation of the classical C3 convertase (9, 11) and fluid phase C3b inhibiting AP activity (12). Furthermore, C4BP can accelerate the decay of formed classical pathway C3 convertase (11).

The success of any disease-causing organism depends on its ability to resist host immunity (13–15). As FH and C4BP are soluble proteins, a wide variety of pathogens have evolved mechanisms to bind and recruit these proteins to their surface, thus disrupting complement deposition (13, 16). Most bacteria recruit FH through CCP domains 6–7 and 18–20, thereby permitting FH domains 1–4 to inhibit complement (10, 15). Pathogen binding of C4BP is generally associated with CCP1-3, which is also responsible for C4b and C3b binding (11, 15). However, C4BP is a multimeric protein with seven identical alpha chains, which permits its simultaneous binding to different ligands while maintaining complement inhibitory activity (11, 15).

Infections are primarily curtailed by antibiotics or vaccines. In recent years, antibiotic resistance has become a major health problem globally, with the proliferation of multidrug-resistant bacteria (17). Development of new antibiotics and vaccines does not appear to meet the current medical demand, therefore new antimicrobial approaches are urgently needed. Chimeric proteins that fuse pathogen binding domains of either FH (CCPs 6–7) of the Fc region of human IgG (FH6–7/IgG) or C4BP (CCPs 1–2) fused to the constant domains CH2, CH3, and CH4 of IgM (C4BP1–2/IgM) resulting in a hexavalent chimeric protein have been developed as alternative strategies to control infection (18, 19). The net result is displacement of complement inhibitors from the bacterial surface with simultaneous complement activation via Fc-C1q interaction, which increases pathogen elimination. This approach has shown promise in enhancing killing of Non-typeable *Haemophilus influenzae* (NTHi) (20), *Neisseria meningitidis* (18), *Neisseria gonorrhoeae* (19, 21), and *Streptococcus pyogenes* (22). In this study we tested the bactericidal activity of both fusion proteins against *M. catarrhalis*.

## Materials and Methods

### Bacteria and Cell Line Culture Conditions

Bacteria used in this study were *M. catarrhalis* strain RH4 (23), isogenic mutants of RH4 devoid of UspA1 (Δ*uspA1*), UspA2 (Δ*uspA2*), and both UspA1 and UspA2 (Δ*uspA1*Δ*uspA2*) (24), and clinical isolates KR473, KR478, KR479, KR485, KR502, KR507, KR508, KR530, KR533, KR488, KR504, KR506, KR531, KR539, KR540, KR477, KR480, KR482, KR510, KR515, and KR527 (25). All strains of *M. catarrhalis* were routinely cultured on chocolate blood agar and grown overnight at 37°C with 5% CO_2_. Prior to experiments, bacteria were sub-cultured from overnight plates and streaked onto new chocolate blood agar plates and grown for 6–8 h. Bacteria were scraped from plates and resuspended into freezing medium [25% brain-heart infusion (BHI)/glycerol], and subsequently aliquoted and stored at −80°C until use.

*Staphylococcus aureus* strain USA300 JE2 were grown in tryptic soy broth at 37°C with shaking (200 rpm). Overnight cultures were normalized to an OD_600 nm_ = 0.1 in fresh medium and grown under the same conditions until an OD_600 nm_ = 0.3–0.4, corresponding to mid-exponential phase of growth. Bacteria were centrifuged and washed once in sterile PBS and normalized to an OD_600 nm_ = 1 which equates to 1–2 × 10^8^ CFU/ml.

FreeStyle Chinese hamster ovary (CHO) S suspension cells (Life Technologies), grown in FreeStyle CHO expression medium supplemented with 8 mM L-glutamine were used for the expression of FH6-7/IgG fusion protein as described (22). Cell cultures were grown in 250 ml Erlenmeyer flasks at 37°C with 8% CO_2_ with shaking (130 rpm). Adherent CHO cells grown in serum-free OptiMEM Glutamax medium were used for the expression of C4BP1-2/IgM as described (19). For expression and collection of C4BP1-2/IgM fusion protein, adherent cells were washed twice in PBS and grown in Opti-MEM for 48 h. Following 48 h, cells were washed and grown in CHO expression medium for 24 h and this procedure repeated for 15 days.

### Purification of Fusion Proteins

Fusion protein FH6-7/IgG were expressed in CHO cells and purified on protein A/G columns as described previously (22). Bound proteins were eluted using 0.1M glycine, pH = 2.7. Protein eluate was dialyzed three time in PBS at 4°C and protein concentrations calculated on aBio photomoter. The C4BP1-2/IgM fusion protein was purified using a HiTrap Normal human serum (NHS)-activated Sepharose 5 ml column coupled with antibody MK104, which recognizes the CCP-1 domain of C4BP as described previously (19).

### Binding of Fusion Proteins to *M. catarrhalis*

*Moraxella catarrhalis* glycerol stocks were thawed at 37°C for 30 min and washed once in PBS for 5 min at 5000 × *g*. Bacteria were normalized to OD_600 nm_ of 1, stained with carboxyfluorescein succinimidyl ester (CFSE, 10 μM) for 30 min at 37°C and washed once in PBS, for gating in flow cytometry assays. Bacterial concentrations were adjusted to an OD_600 nm_ of 0.5 in PBS and thereafter 50 μl was mixed with 50 μl of increasing concentrations of fusion proteins for 30 min at 37°C in a covered V-bottomed 96 well plate. Bacteria were centrifuged for 5 min at 5000 × *g* at room temperature (RT) and washed once in PBS. To detect FH6-7/IgG binding to *M. catarrhalis*, bacteria were stained with Alexa Fluor (AF) – 488 goat anti-human IgG (1:1000; Invitrogen) in 1% (w/v) BSA/PBS for 30 min at RT. To detect C4BP1-2/IgM binding, bacteria were stained with polyclonal rabbit anti-human IgM (1:1000; Dako) for 30 min at RT. Bacteria were centrifuged and washed once in 1% (w/v) BSA/PBS and for C4BP1-2/IgM detection, followed by a secondary antibody staining (AF-647 goat anti-rabbit, 1:1000; Invitrogen) for 30 min in the dark. For both fusion proteins, following Ab staining, bacteria were centrifuged and washed once in 1% (w/v) BSA/PBS, resuspended in 150 μl PBS and deposited fusion proteins were assessed using a CytoFLEX flow cytometer. Bacteria incubated without fusion protein was used as a negative control. CFSE- stained and non-stained bacteria were used for gating purposes and a minimum of 20,000 events were examined. To assess the binding of C4BP-IgM in the presence of serum, the fusion protein was labeled with AF 488 using AF 488 microscale labeling kit (A10235, Molecular Probes).

### Serum Bactericidal Assays

Bacteria were prepared as described in bacteria-fusion protein binding section, with the omission of the CFSE staining. NHS was prepared from freshly drawn blood with informed consent from at least eight healthy volunteers as described (26) and in accordance with the recommendations of the ethical committee at Lund University (Permit 2017/582) and the Declaration of Helsinki (27). Bacteria were normalized to OD_600 nm_ of 0.05. Fifty microliters of bacteria were incubated in the presence or absence of 40 μl of fusion protein (final concentration 50 μg/ml) for 30 min at 37°C in GVB^++^ buffer [5 mM veronal buffer pH 7.3, 0.1% (w/v) gelatin, 140 mM NaCl, 1 mM MgCl_2_, and 0.15 mM CaCl_2_]. After 30 min, 10 μl of pooled NHS was added and incubated with bacteria for a further 30 min at 37°C. Alternatively, bacteria and fusion protein in GVB^++^ buffer (0–100 μg/ml) in addition to serum were added simultaneously and incubated for 30 min at 37°C. For calculation of bacteria survival, samples of bacteria were removed at time 0 and time 30 min following incubation at 37°C, serially diluted in PBS and spread onto chocolate agar plates for colony enumeration after growth overnight at 37°C with 5% CO_2_. As a control heat-inactivated serum was used following treatment at 56°C for 30 min.

### Binding Competition Experiments

Bacteria were thawed, washed once in PBS and normalized to an OD_600 nm_ of 0.5. Two experimental protocols were utilized: (1) Bacteria were pre-incubated with varying concentrations of either FH6–7/IgG or C4BP1–2/IgM in GVB^++^ buffer for 30 min at 37°C. Following incubation, NHS treated with the complement C5 inhibitor OmCI (10 μg/ml; 0.625 μM) (expression vector obtained from Swedish Orphan Biovitrum) (28) on ice for 30 min was added to a final concentration of 10% and incubated at 37°C for 30 min; (2) Bacteria, FH6-7/IgG fusion protein (at a final concentration of 0, 50, or 100 μg/ml) and OmCI-treated NHS (final concentration of 10%) were added simultaneously and incubated at 37°C for 30 min. Following incubation, bacteria were centrifuged and washed once in 1% (w/v) BSA/PBS. To determine serum FH binding, biotinylated Ab specific to CCP domain 5 of FH (MRC OX-24-biotin; 1:500) was used for 30 min at RT. Bacteria were washed once in 1% (w/v) BSA/PBS and stained with AF647-streptavidin (1:1000; Invitrogen) in the dark for 30 min at RT. To determine serum C4BP binding, Ab specific to CCP domain 4 of the alpha chains of C4BP (MK67; 1:1000) (29) was used for 30 min at RT. Bacteria were washed once in 1% (w/v) BSA/PBS and stained with AF-647 goat anti-mouse (1:1000; Invitrogen). Bacteria were centrifuged and washed once in 1% (w/v) BSA/PBS, resuspended in 150 μl PBS and serum FH or C4BP binding were assessed using a CytoFLEX flow cytometer.

### Complement Deposition Assays

Bacteria were prepared as described in the binding assay section. For detection of complement components C3d and iC3b, bacteria, fusion protein, and OmCI-treated NHS (final concentration of 5%) were incubated for 30 min at 37°C. Bacteria were centrifuged and washed once in PBS and stained with either a monoclonal murine anti-human C3d Ab (1:1000, Quidel, A207) or a monoclonal murine anti-human iC3b Ab (1:1000, Quidel, A209) in 1% (w/v) BSA/PBS for 30 min at RT. For detection of MAC, NHS at a final concentration of 10% was used and bacteria incubated for 20 min at 37°C. Following washing, bacteria were stained with monoclonal mouse anti-human C9 neoantigen (1:1000, Hycult, aE11) for 30 min at RT. Bacteria were centrifuged and washed once in PBS followed by staining with AF-647 goat anti-mouse, 1:1000; Invitrogen) for 30 min in the dark at RT. Bacteria were centrifuged and washed once in PBS, resuspended in 150 μl of PBS and deposited complement components detected using a CytoFLEX flow cytometer (Beckman Coulter).

### Whole Blood Survival and Phagocytosis Assay

*Moraxella catarrhalis* were prepared as described in the binding assay section. *S. aureus* were grown as described on the bacteria section. Bacteria were normalized to OD_600 nm_ of 1, stained with CFSE (10 μM) for 30 min at 37°C and washed once in PBS.

Human blood was taken from healthy volunteers and treated with lepirudin (Refludan 50 μg/ml; Celgene). Five hundred microliters of blood were incubated for 30 min with 1 μM OmCI, 10 μM cytochalasin D (Sigma-Aldrich) or both, on an end-over-end shaker at 37°C and 5% CO_2_. Approximately 5 × 10^5^ CFU of CFSE-stained bacteria were added to blood, and blood suspensions were incubated on a shaker at 37°C and 5% CO_2_.

To assess the survival of bacteria in blood, at specific time points a sample of the suspension was collected, serially diluted in PBS and plated onto chocolate agar plates (*M. catarrhalis*) or blood agar plates (*S. aureus*). Agar plates were incubated overnight at 37°C and 5% CO_2_ and CFU/mL determined following enumeration of surviving bacteria.

To assess the phagocytosis of bacteria, at specific time points a sample of the suspension was collected, red blood cells lysis was performed by addition of water followed by addition of 10× PBS to restore osmotic pressure. Cells were centrifuged at 300 *g* for 5 min and fixed by incubation in 4% paraformaldehyde in PBS for 15 min at RT. Cells were stained with anti-CD14 APC antibody (Beckman Coulter) for 30 min, washed once in PBS and resuspended in PBS for analysis using a CytoFLEX flow cytometer.

### Statistical Analysis

To examine differences between experimental groups, a one-way or two-way ANOVA was used (GraphPad Prism v7.0); a *p*-value was <0.05 was considered statistically significant. The *p*-values shown in figure legends represent the *post hoc* tests.

## Results

### Fusion Proteins Bind to *M. catarrhalis*

**FIGURE 1:**
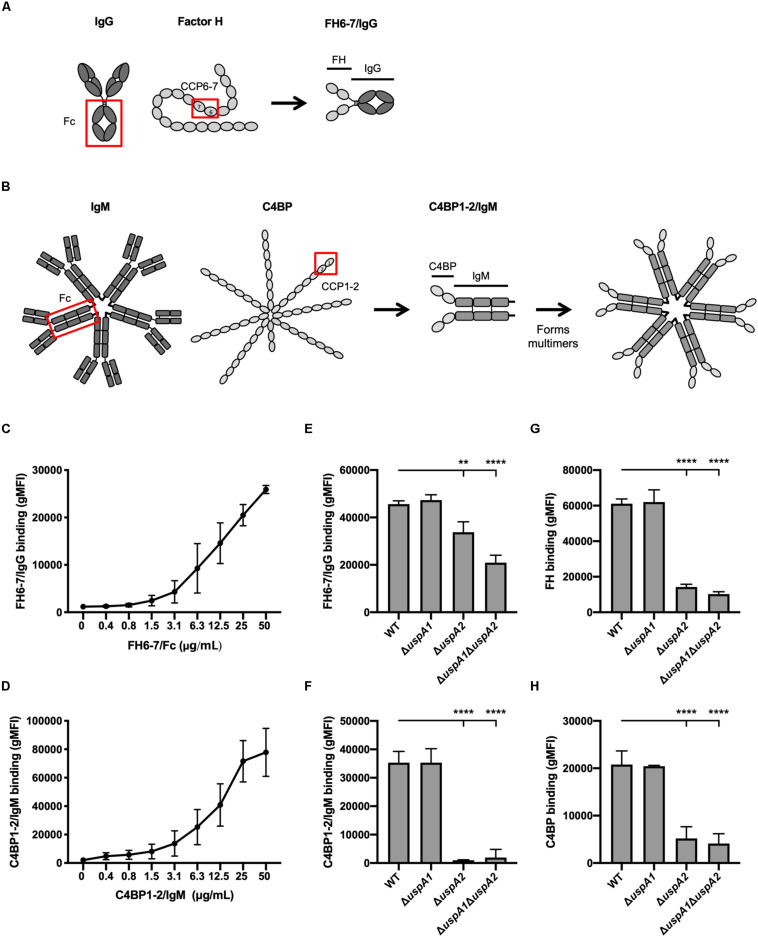
FH 6-7/IgG and C4BP1-2/IgM bind to *M. catarrhalis*. **(A,B)** Schematic representation of fusion proteins FH6-7/IgG and C4BP1-2/IgM, respectively. **(C)** Binding of FH6-7/IgG and **(D)** C4BP1-2/IgM to *M. catarrhalis* RH4 [wild-type (WT) reference strain] assessed by flow cytometry. **(E–H)** Binding of RH4 WT and isogenic mutants lacking *uspA1* and/or *uspA2* to **(E)** FH6-7/IgG, **(F)** C4BP1-2/IgM, **(G)** factor H (FH), and **(F)** C4b-binding protein (C4BP). Mean (±SD) from three independent experiments are shown. Statistical significance of differences was calculated using one-way ANOVA with Dunnett’s post-test **(E–H)**; **p < 0.01 and ****p < 0.0001.

The fusion protein FH6–7/IgG was created by combining the CCP6 and CCP7 domains of FH with the Fc region of human IgG (Figure 1A). C4BP1–2/IgM was made by fusing the CCP1 and CCP2 domains of C4BP together with the constant domains CH2, CH3, and CH4 of human IgM (Figure 1B). C4BP1–2/IgM forms multimers similarly to IgM, hexamers (dodecamers of C4BP CCPs 1–2; ∼80%) or pentamers (decamers of C4BP CCPs 1–2; ∼20%) (19).

To understand whether fusion proteins (FH6–7/IgG and C4BP1–2/IgM) could be used as alternative treatment strategies, we first explored whether these proteins could bind the well-characterized *M. catarrhalis* strain RH4. *M. catarrhalis* is known to bind both soluble complement inhibitors, FH and C4BP (24, 30). Fusion proteins incubated with bacteria over a range of concentrations confirmed that *M. catarrhalis* interacts with FH6–7/IgG (Figure 1C) and C4BP1–2/IgM (Figure 1D) in a dose-dependent fashion.

The ubiquitous surface proteins (Usp) A1 and A2 are high-molecular weight proteins that are abundantly expressed at the surface of *M. catarrhalis* (31). UspA2 plays an important role in immunity evasion as it can bind the complement inhibitors C4BP (24), vitronectin (32) and plasminogen (33). To test the possibility that the fusion proteins bind *M. catarrhalis* through UspA1 and/or UspA2, we used mutants lacking UspA1 and/or UspA2. The binding of FH6–7/IgG was reduced in the single mutant Δ*uspA2* and the double mutant Δ*uspA1*Δ*uspA2*, but not in Δ*uspA1*, compared to the wild-type (WT) strain (Figure 1E). A similar observation was made when assessing the binding of whole FH (Figure 1G), suggesting that the fusion protein FH6–7/IgG, like FH, binds partly through UspA2. The binding of C4BP1–2/IgM was unchanged in Δ*uspA1* compared to WT *M. catarrhalis*, but almost completely abrogated in Δ*uspA2* and Δ*uspA1*Δ*uspA2* (Figure 1F), similar to the binding of C4BP from NHS (Figure 1H). Therefore, the fusion protein C4BP1–2/IgM binds *M. catarrhalis* mainly through UspA2.

### FH6–7/IgG but Not C4BP1–2/IgM Prevents Binding and Outcompetes Respective Soluble Complement Inhibitor

**FIGURE 2:**
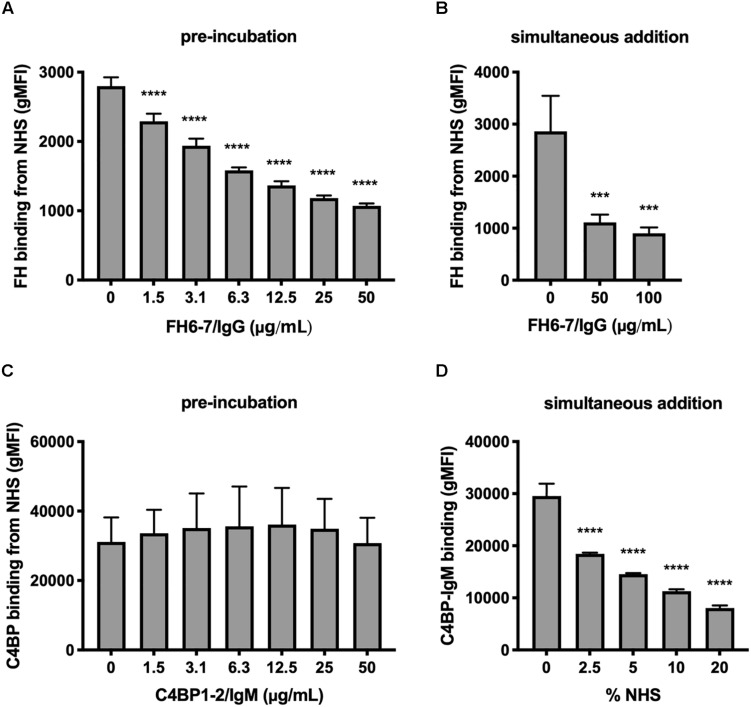
FH6-7/IgG competes out serum FH, but C4BP1-2/IgM does not compete out serum C4BP. **(A)** *M. catarrhalis* were pre-incubated in increasing amounts of FH6-7/IgG, followed by the addition of OmCI-treated serum (C5-inhibitor). The binding of FH from NHS was assessed by flow cytometry using antibodies specific to domains present in serum derived FH but absent on the fusion proteins. **(B)** FH6-7/IgG and NHS were added simultaneously to the bacteria and binding of FH from NHS was assessed. **(C)** *M. catarrhalis* pre-incubated in increasing amounts of C4BP1-2/IgM, followed by OmCI-treated serum were tested for the binding of C4BP from NHS by flow cytometry using antibodies specific to domains present in serum derived C4BP but absent on the fusion proteins. **(D)** Alexa Fluor 488-labeled C4BP-IgM was added simultaneously with differing concentrations of OmCI-treated serum to bacteria to assess the binding of the fusion protein. Mean (±SD) from three independent experiments are shown. Statistical significance of differences was calculated using one-way ANOVA with Dunnett’s post-test; ***p < 0.001 and ****p < 0.0001.

We investigated the interplay between fusion proteins and the ability of *M. catarrhalis* to recruit FH and C4BP to the bacterial surface. Firstly, we pre-incubated *M. catarrhalis* strain RH4 with increasing concentrations of FH6–7/IgG or C4BP1–2/IgM followed by the addition of OmCI-treated serum as a source of FH and C4BP. After incubation we examined the amount of FH or C4BP binding with antibodies specific to CCP domains present in serum-derived FH and C4BP, but not in the respective Fc fusion proteins. Preincubation of RH4 with FH6–7/IgG with concentrations as low as 1.5 μg/ml resulted in a significant reduction in FH binding from serum with an inverse correlation between FH6–7/IgG and FH binding continuing in a dose-dependent fashion (Figure 2A). Next, we assessed whether FH6–7/IgG could outcompete serum FH in binding to *M. catarrhalis* when added concurrently with NHS. Under these experimental settings, FH6–7/IgG also outcompeted FH binding from serum (Figure 2B). In contrast, no difference was observed in serum C4BP binding when bacteria were pre-incubated with increasing concentrations of C4BP1–2/IgM (Figure 2C). These data show that FH6–7/IgG can successfully displace FH at the surface of *M. catarrhalis*, while C4BP1–2/IgM does not out-compete serum C4BP, suggesting that C4BP binds the bacteria with a higher affinity than the fusion protein. Based on the observation that C4BP-IgM does not outcompete C4BP for binding to the bacteria, we tested whether C4BP-IgM could bind bacteria in the presence of serum (OmCI-treated). We found that the binding of C4BP-IgM is decreased when increasing concentrations of serum are added at the same time (Figure 2D), but there seems to be still a significant binding in the presence of 10% NHS which is the amount used in serum bactericidal assays.

### Serum Bactericidal Activity Is Enhanced by Fusion Proteins

**FIGURE 3:**
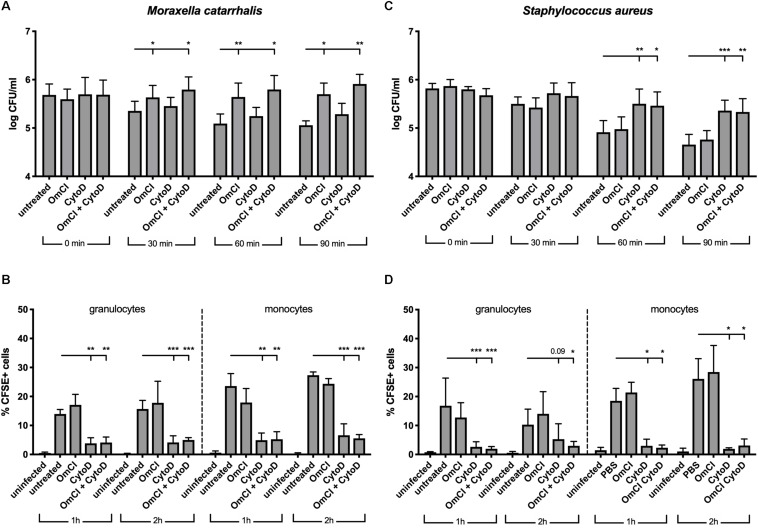
In a whole blood model, *M. catarrhalis* is killed through MAC-mediated killing. CFSE-labeled bacteria were added to whole blood previously incubated with 1 μM OmCI, 10 μM cytochalasin D or both. **(A,B)** *M. catarrhalis* clinical isolate #473 was picked as more serum resistant than most clinical isolates and the reference strain RH4 **(C,D)** *Staphylococcus aureus* JE2 was used as an example of MAC-resistant bacteria **(A,C)** Survival of bacteria overtime was assessed by collecting a sample at *t* = 0, 30, 60, and 90 min post-infection, and plating bacteria on agar plates. The number of bacteria is represented as log of CFU/ml. **(B,D)** Phagocytosis was assessed by detection of CFSE+ cells among granulocytes and monocytes from whole blood. At 1 and 2 h post-infection, a sample was collected, red blood cells were lysed, cells were fixed and stained with anti-CD14, and cells were analyzed using flow cytometry. The percentage of CFSE+ cells was measured in granulocytes and monocytes (gated using Side Scatter and CD14 staining). Mean (±SD) from four **(A)** or three **(B–D)** independent experiments are shown. Statistical significance of differences was calculated using two-way ANOVA with Dunnett’s post-test; *p < 0.05; **p < 0.01; and ***p < 0.001.

Complement activation results in phagocytic clearance of bacteria as well as direct bacterial killing through the formation of lytic MAC. Gram-positive bacteria are not sensitive to MAC-mediated killing, but it plays a critical role against Gram-negative bacteria such as *M. catarrhalis* (34). In order to assess the respective roles of phagocytosis and MAC-mediated killing in clearance of *M. catarrhalis*, we employed a whole blood infection model, using human blood pre-treated with complement C5 inhibitor OmCI, phagocytosis inhibitor cytochalasin D or both. We observed that *M. catarrhalis* survival in whole blood is increased when complement is inhibited but not significantly changed when phagocytosis was inhibited (Figure 3A). However, phagocytosis of *M. catarrhalis* by granulocytes and monocytes remained unchanged by OmCI compared to untreated blood, while cytochalasin D inhibited phagocytosis as expected (Figure 3B). It is worth noting that this method does not distinguish between live and dead bacteria being taken up by phagocytes. This confirms that phagocytosis does not play a major role in *M. catarrhalis* killing in whole blood as it does not correlate with bacteria survival numbers: bacteria survive much better in OmCI-treated blood than untreated blood, however phagocytosis is unchanged. To confirm that our model works, and to highlight the difference between Gram-negative *M. catarrhalis* and a Gram-positive bacteria, we performed the same experiments in parallel with *S. aureus*. As expected, inhibition of phagocytosis increased the survival of *S. aureus*, while complement inhibition at the level of C5 did not (Figure 3C). Phagocytosis of *S. aureus* was similar to *M. catarrhalis*, as it was unchanged by OmCI but inhibited by cytochalasin D (Figure 3D). Altogether, this experiment confirms in an environment containing both complement and phagocytes, *M. catarrhalis* is killed through MAC-mediated lysis. Based on this, the effect of the fusion proteins in killing *M. catarrhalis* will be evaluated in serum alone.

**FIGURE 4:**
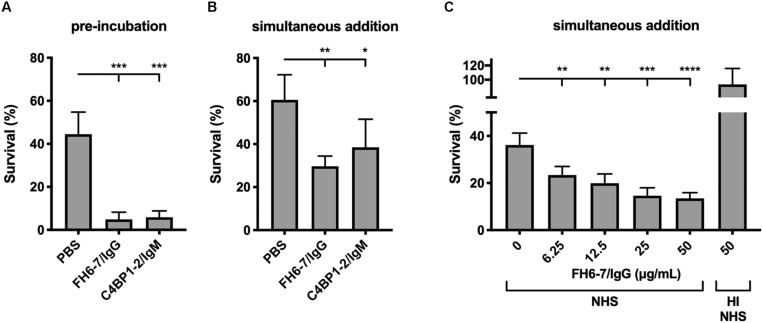
FH6-7/IgG and C4BP1-2/IgM increase serum killing of *M. catarrhalis*. **(A)** Bacteria were pre-incubated with PBS (control), 50 μg/ml of FH6-7/IgG or 50 μg/ml of C4BP1-2/IgM for 30 min before addition of 10% NHS. Bacteria were counted at *t* = 0 and *t* = 30 min post-addition of serum and survival percentage was calculated. **(B)** Fusion proteins and serum were added simultaneously to the bacteria, and bacteria survival was calculated. **(C)** Increasing amounts of FH6-7/IgG were added simultaneously with NHS to bacteria and survival was calculated. Heat-inactivated NHS was used as a negative control of bacteria killing. Mean (±SD) from three **(A,C)** or four **(B)** independent experiments are shown. Statistical significance of differences was calculated using one-way ANOVA with Dunnett’s post-test; *p < 0.05; **p < 0.01; ***p < 0.001; and ****p < 0.0001.

Recruitment of FH and or C4BP to the bacterial surface interferes with complement deposition and contributes to serum resistance. Therefore, we tested the ability of FH6–7/IgG and C4BP1–2/IgM to augment serum killing. Both fusion proteins, when pre-incubated with *M. catarrhalis* at a final fusion protein concentration of 50 μg/ml, resulted in a significant reduction in bacterial survival (Figure 4A). C4BP1–2/IgM did not displace serum C4BP (Figure 2C), but still bound bacteria in the presence of serum (Figure 2D), and the hexavalent structure of the C4BP1–2/IgM fusion protein would most likely be a potent activator of the classical pathway of complement, explaining the reduced bacterial survival compared to no protein serum control. Regarding the enhanced FH6–7/IgG-dependent serum killing, both displacement of FH from the bacterial surface and Fc-mediated activation of the classical pathway were likely to have contributed to enhanced killing.

When fusion proteins were added with bacteria and serum simultaneously we observed a statistically significant decrease in survival with FH6–7/IgG and C4BP1–2/IgM (Figures 4A,B). However, the reduction is less apparent with C4BP1–2/IgM compared to FH6–7/IgG, likely because the C4BP1–2/IgM fusion protein does not prevent serum C4BP binding on the bacterial surface. Finally, we confirm that FH6–7/IgG enhances serum killing of strain RH4 in a dose-dependent fashion that requires active complement (Figure 4C).

### FH6–7/IgG Enhances C3b and MAC Deposition

**FIGURE 5:**
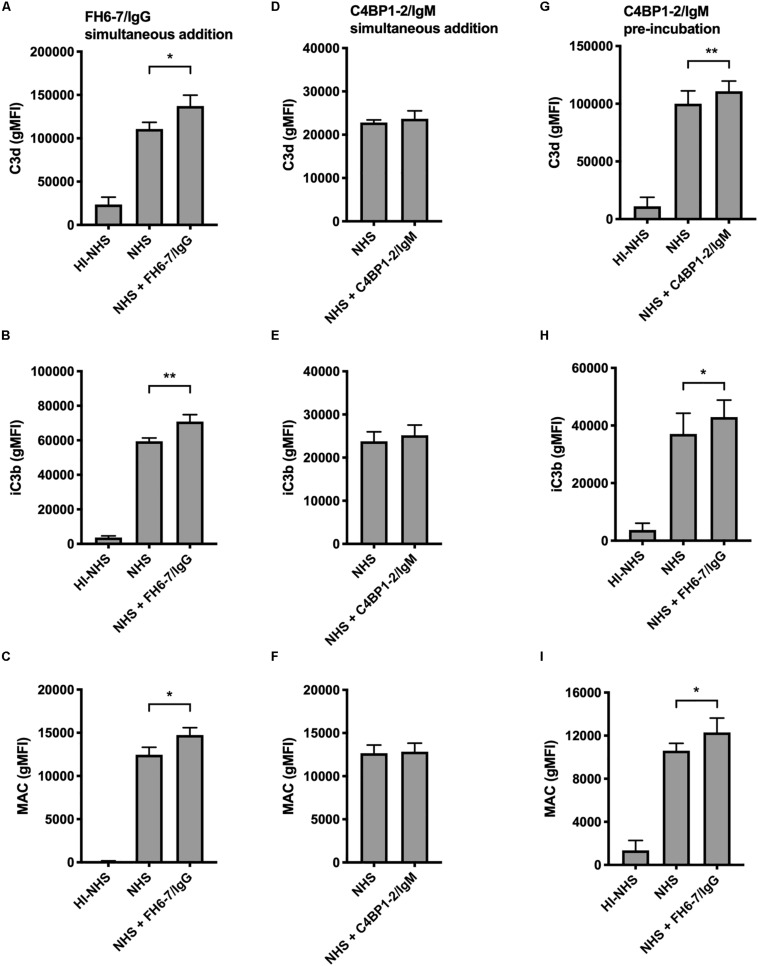
Fusion proteins increase complement deposition on the bacterial surface. Flow cytometry was used to measure C3d, iC3b, and MAC binding at the surface of *M. catarrhalis*. **(A–C)** FH6-7/IgG and serum were added simultaneously to bacteria. Bacteria were incubated with 5% OmCI-treated serum for 30 min for C3 deposition, or 10% NHS for 20 min for MAC deposition. Heat-inactivated serum was used as a negative control of complement activation. **(D–F)** C4BP1-2/IgM and serum were added simultaneously to bacteria. **(G–I)** Bacteria were pre-incubated with C4BP1-2/IgM before addition of serum. Mean (±SD) from three **(A–F)** or four **(G–I)** independent experiments are shown. Statistical significance of differences was calculated using one-way ANOVA with Dunnett’s post-test; *p < 0.05; **p < 0.01.

In order to understand how FH6–7/IgG increased serum sensitivity of *M. catarrhalis* we examined the deposition of complement components iC3b, C3d, and MAC in the presence and absence of fusion protein using flow cytometry. We choose to investigate both iC3b and C3d as markers of C3b deposition due to our previous experience with non-specific binding and anomalies with Ab detection of cleaved complement components. In accordance with the serum bactericidal data, the presence of FH6–7/IgG resulted in a significant increase in iC3b, C3d, and MAC deposition compared to no FH6–7/IgG control (Figures 5A–C). In contrast, no significant differences in complement deposition were observed when C4BP1–2/IgM was simultaneously added with serum, likely because this assay is not sensitive enough to detect a smaller increase in complement deposition (Figures 5D–F). However, an increase in complement deposition is observed when bacteria were pre-treated with C4BP1–2/IgM, in accordance with the increase in serum killing (Figures 5G–I).

### FH6–7/IgG Binds a Broad Panel of *M. catarrhalis* Clinical Isolates Resulting in Enhanced Serum Killing

**FIGURE 6:**
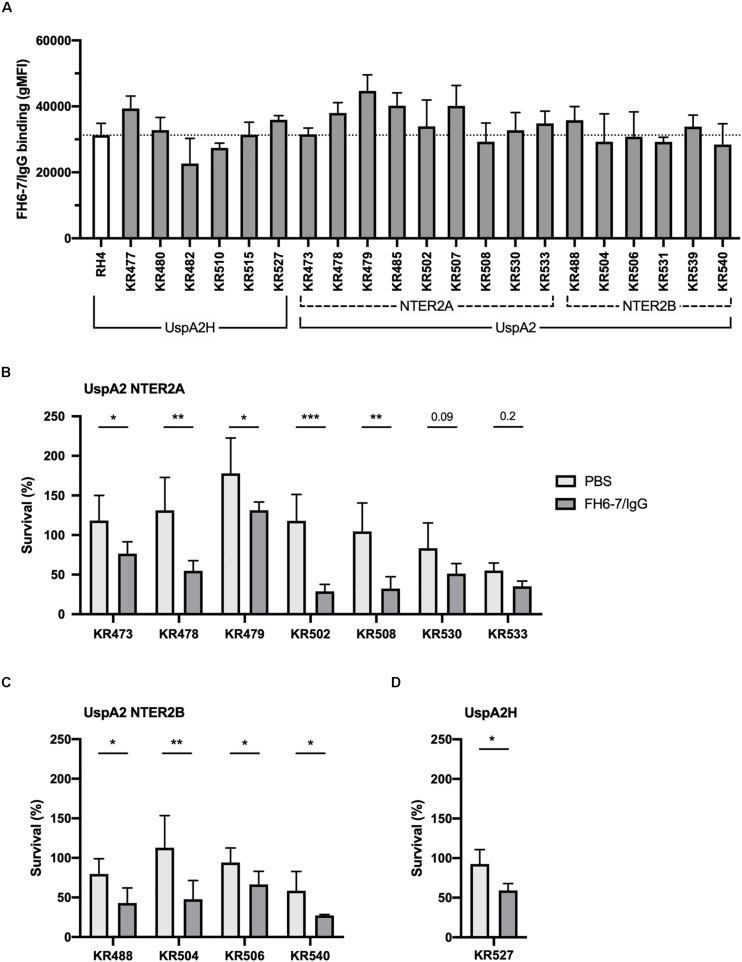
FH6-7/IgG binds to and increases serum killing of a large majority of *M. catarrhalis* clinical isolates. **(A)** 21 clinical isolates and the laboratory strain RH4 were tested for FH6-7/IgG binding by flow cytometry. In the absence of FH6-7/IgG, bacteria incubated with detection antibody had a similar gMFI for all isolates (average 151, standard deviation 45). **(B–D)** Twelve clinical isolates were treated with 50 μg/ml of FH6-7/IgG or PBS, at the same time as 15% NHS, and their survival after 30 min was assessed by plating bacteria and counting remaining colony forming-units (CFU). The remaining nine isolates were not included because their survival in serum in the absence of fusion protein was less than 5%. **(B)** UspA2 NTER2A strains, **(C)** UspA2 NTER2B strains, **(D)** UspA2H strains. Mean (±SD) from three **(A–C)** or four **(D)** independent experiments are shown. Statistical significance of differences was calculated using two-way ANOVA with Holm–Sidak’s post-test; *p < 0.05; **p < 0.01; ***p < 0.001.

To ascertain whether FH6–7/IgG could be used as a novel immunotherapeutic we investigated the activity of this fusion protein against a panel of genetically diverse clinical isolates of *M. catarrhalis*. These isolates represent a broad diversity of virulent *M. catarrhalis* strains, and several UspA types: UspA2H (like RH4), UspA2 NTER2A or NTER2B. Both fusion proteins bind RH4 through the adhesin UspA2 (Figures 1E,F), therefore it is of interest to know whether different UspA2 types can also bind FH6–7/IgG and C4BP1–2/IgM. All *M. catarrhalis* clinical isolates bound FH6–7/IgG, similarly to our lab strain RH4, regardless of their UspA type (Figure 6A). This binding of FH6–7/IgG fusion protein correlated with increased serum killing of a majority of the *M. catarrhalis* clinical isolates independently of their UspA type, when fusion protein and serum were added simultaneously (Figures 6B–D). This shows that FH6–7/IgG could constitute a promising therapeutic strategy in a large number of *M. catarrhalis* infections.

### C4BP1–2/IgM Binds a Broad Panel of *M. catarrhalis* Clinical Isolates Resulting in Enhanced Serum Killing

**FIGURE 7:**
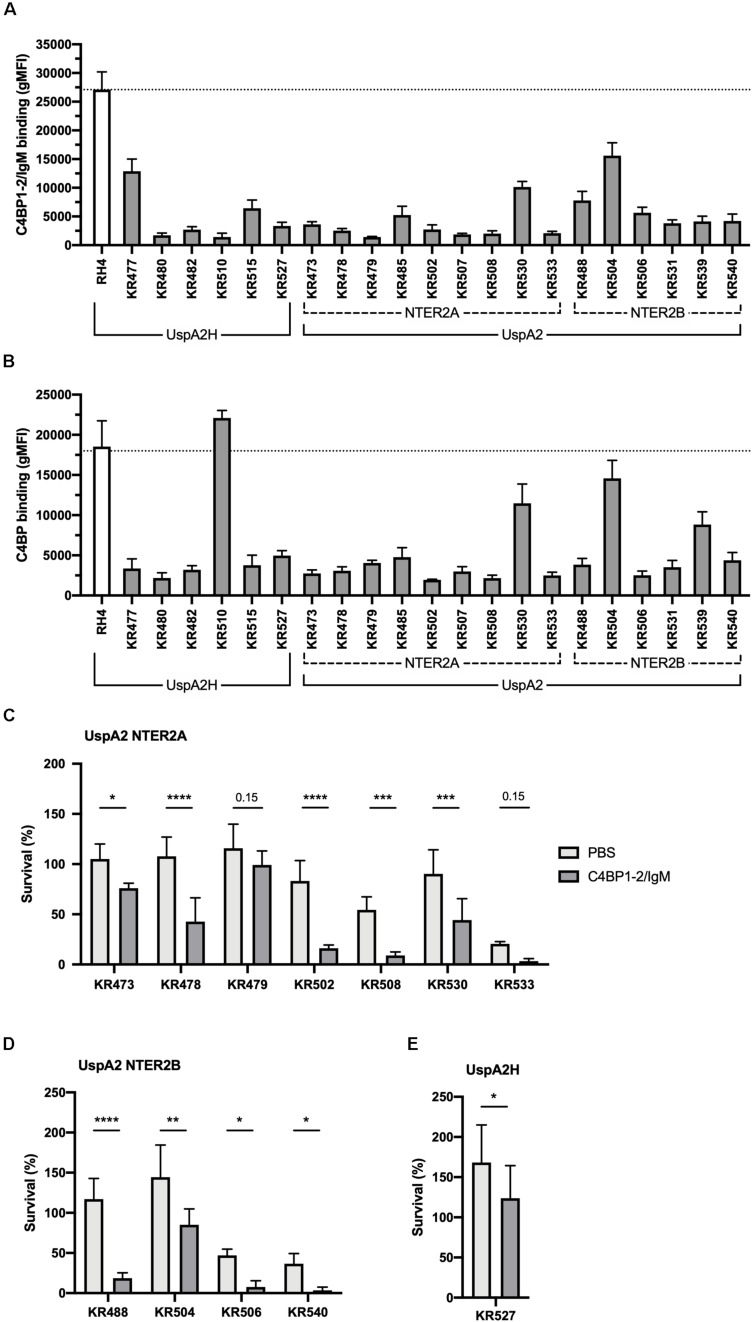
C4BP1-2/IgM binds to and increases serum killing of many of the *M. catarrhalis* clinical isolates tested. **(A)** 21 clinical isolates and the lab strain RH4 were tested for C4BP1-2/IgM binding by flow cytometry. The gMFI for control without C4BP1-2/IgM was similar in all isolates (average 152, SD 75). **(B)** Clinical isolates were tested for C4BP binding from NHS by flow cytometry. Moraxella isolates were treated with 5% NHS + OmCI for 30 min. The gMFI for control without NHS was similar in all isolates (average 207, SD 132). **(C–E)** Twelve clinical isolates were treated with 50 μg/ml of C4BP1-2/IgM or PBS, before addition of as 15% NHS, and their survival after 30 min was assessed by plating bacteria and counting remaining CFU. The remaining nine isolates were not included because their survival in serum in the absence of fusion protein was less than 5%. **(C)** UspA2 NTER2A strains, **(D)** UspA2 NTER2B strains, **(E)** UspA2H strains. Mean (±SD) from three **(A)** or four **(B–E)** independent experiments are shown. Statistical significance of differences was calculated using two-way ANOVA with Holm–Sidak’s post-test; *p < 0.05; **p < 0.01; ***p < 0.001; ****p < 0.0001.

Similarly, we next investigated the activity of C4BP1–2/IgM against a collection of genetically diverse *M. catarrhalis* clinical isolates. Surprisingly, all clinical isolates bound C4BP1–2/IgM much less than our reference strain RH4 (Figure 7A). To test whether this lower binding could be due to the fact that these isolates bind C4BP less than RH4, we assessed the binding of C4BP from serum to these isolates. With the exception of KR510, we observed significant correlation between the binding of C4BP1–2/IgM and C4BP from serum (Figure 7B). In addition all clinical isolates bound C4BP less than the laboratory strain RH4 (Figure 7B). Although KR510 bound the fusion protein poorly, it bound C4BP very strongly, suggesting that it may bind C4BP through domains other than CCP1 and 2, the only C4BP domains present in the fusion protein. Based on the following observations; (1) C4BP1–2/IgM cannot prevent the binding of C4BP from serum; (2) *M. catarrhalis* clinical isolates bound C4BP1–2/IgM more poorly than RH4; and (3) C4BP1–2/IgM was less efficient at enhancing RH4 serum killing when simultaneously added (Figure 4B); we investigated serum survival of clinical isolates pre-treated with C4BP1–2/IgM. Although clinical isolates bound C4BP1–2/IgM more poorly than RH4, we observed an increased killing of the majority of *M. catarrhalis* isolates when they were pretreated with C4BP1–2/IgM (Figures 7C–E).

## Discussion

The common respiratory pathogen *M. catarrhalis* causes significant morbidity and economic burden because it is responsible for otitis media in children and exacerbations of chronic obstructive pulmonary disease in older individuals (2, 3). Currently, there is no vaccine to prevent *M. catarrhalis* infection. Given that otitis media is the most common reason for which children receive antibiotics (35), treatment of *M. catarrhalis* disease with antibiotics promotes antibiotic resistance and also disrupts normal commensal microbiota (1). Therefore, the development of novel adjunctive anti-infective immunotherapeutics against opportunistic pathogens such as *M. catarrhalis* would be highly beneficial.

Complement is present and active in both anatomical regions in which *M. catarrhalis* causes disease. In the ear, complement cleavage activation fragments of C3, C4, and Factor B have been detected in patients with otitis media with effusions (36). In a separate study, the cytolytic activity and presence of the MAC was observed in middle ear effusions obtained from persistent otitis media infections (37). In addition, C3-coated bacteria were identified in a small number of middle ear exudates originating from children suffering from acute otitis media (38). The role of complement in preventing middle ear infection has been demonstrated. Complement depletion by use of cobra venom factor restored the capacity of two avirulent NTHi strains to cause otitis media in a chinchilla model of infection (39). In the lungs, local synthesis of complement occurs; pulmonary alveolar type II epithelial cells secrete complement proteins C2, C4, C5, and Factor B (40), while bronchiolar epithelial cells generate C3 and membrane bound complement regulatory proteins (41). Complement components have been detected in bronchial secretions isolated from guinea pigs. Importantly, enhanced titers of complement proteins were observed following intranasal infection of guinea pigs with mycoplasma, indicating a role for complement in tackling lung infections (42). Moreover, intranasal administration of FH6–7/IgG enhanced clearing of NTHi in a mouse model of lung infection highlighting the therapeutic potential of this fusion protein (20).

In this study, we showed that fusion proteins, FH6–7/IgG and C4BP1–2/IgM, constitute novel antimicrobial strategies effective in eliminating *M. catarrhalis*. In particular, FH6–7/IgG bound all tested *M. catarrhalis* clinical strains taken from a cohort of genetically diverse isolates. Importantly, FH6–7/IgG significantly increased serum killing of the vast majority of tested isolates. Our data suggests that enhanced killing was due to a combination of (i) preventing the recruitment of the soluble complement inhibitor FH to the bacterial surface via displacement, while (ii) simultaneously promoting complement deposition by activation of the classical pathway via the exposed IgG Fc region.

Incubation of *M. catarrhalis* with C4BP1–2/IgM also resulted in decreased bacterial survival, although this was less efficient than FH6–7/IgG when added simultaneously with NHS. This is most likely due to the inability of C4BP1–2/IgM to out-compete and displace C4BP recruited from serum. We showed that C4BP1–2/IgM interacts with *M. catarrhalis* through UspA2, similarly to C4BP and FH. C4BP is a large glycoprotein containing seven identical alpha chains housing CCP domains, which are integral for complement inhibition (43). Previous work has illustrated that the binding region responsible for UspA2-C4BP interaction is localized to the CCP2, CCP5, and CCP7 domains of the alpha chain (24). We show that *M. catarrhalis* has the capacity to concomitantly bind C4BP and C4BP1–2/IgM. As a result, bound C4BP can continue to inhibit complement activation in the presence of C4BP1–2/IgM, thus preventing significant bacterial killing. Our results suggest that the bactericidal activity of C4BP1–2/IgM depends on which factor, C4BP1–2/IgM or serum derived C4BP, interacts first with the pathogen *in vivo*, as pre-incubation of the bacterium with C4BP1–2/IgM significantly accelerates serum killing.

Importantly, previous work has shown using *ex vivo* assay and *in vivo* animal models that these fusion proteins do not cause unwanted complement activation or tissue damage. Bettoni et al. (19) confirmed that C4BP1–2/IgM does not deposit complement on human erythrocytes or apoptotic cells. Importantly, this fusion protein lacks the CCP3 domain of C4BP which is required for binding to C4b fragments on the cell surface and therefore interaction of C4BP1–2/IgM with C4b-decorated cells is prevented. Furthermore, the authors showed that C4BP1–2/IgM was effective in preventing gonococcal colonization in a mouse vaginal colonization model using human FH/C4BP transgenic mice. Importantly, no adverse side effects following administration of C4BP1–2/IgM to mice was observed over the duration of the experiment. Blom et al. (22) confirmed that FH6–7/IgG does not enhance classical or AP activation measured via sensitized sheep and rabbit erythrocyte hemolysis assays. In addition, using a platelet aggregation assay, FH6–7/IgG did not activate platelets and no degree of coagulation activation was observed. In addition, FH6–7/IgG was effective in reducing mortality in a streptococcal sepsis model of infection using human FH/C4BP transgenic mice and showed no adverse side effects following administration. Specifically the authors noted no enhanced complement deposition in tissues sections taken from the eyes and kidneys of FH6-7/IgG treated and untreated animals, either infected or not with *S. pyogenes* (22). Lastly, Shaughnessy et al. (44) also observed no short term renal (increases in creatinine) or hematologic (increases in lactate dehydrogenase or decrease in hematocrit) or adverse side effects following systemic administration of FH6–7/IgG. Although this data is encouraging, future toxicology assessments are required and will take place during the preclinical development of these fusion proteins.

Alternative treatments such as fusion proteins could reduce the use of antibiotics, which are associated with many adverse effects such as the development of antibiotic resistance and the destruction of normal respiratory tract microbiota. Importantly, developing new treatment approaches based on components used by bacteria to confer serum resistance will ease or eliminate the selective pressure required to drive resistance against these therapeutics. We show that binding of both fusion proteins (FH6–7/IgG and C4BP1–2/IgM) to *M. catarrhalis* relies heavily on the expression of Usp proteins, particularly UspA2. UspA2 is a non-fimbrial surface protein (45), highly conserved across disease isolates (46) and expressed during *in vivo* infection (47). Resistance to fusion proteins would require the emergence of UspA2 null mutants or UspA2 mutants unable to interact with FH6–7 and/or C4BP1–2 domains of circulating FH and C4BP, respectively, resulting in the reduced ability to recruit these soluble regulators. UspA2 plays even more roles in complement evasion as in addition to FH and C4BP, it also binds vitronectin, a multifunctional glycoprotein which can prevent the formation of MAC on the bacterial surface (32), and plasminogen, a zymogen which when converted to plasmin can degrade central complement components (33). Interaction of UspA2 with these glycoproteins adds extra pressure to maintain the WT UspA2 protein sequence and/or conformation. An inability to bind C4BP, FH, vitronectin and plasminogen due to loss of UspA2 would pose a significant fitness disadvantage promoting eradication by complement activity (24, 32, 48, 49).

*Moraxella catarrhalis* is both a normal commensal and a pathogen of the respiratory tract (50), and although little is known about the differences between the two or how it switches from one to the other, preserving the healthy microbiota when targeting pathogenic bacteria is critical. Microbial community composition has a profound impact on human health, protecting against the growth and invasion of respiratory pathogens (51), helping with the development and maintenance of a healthy immune system (52), but also preventing neurological diseases such as Parkinson’s disease, Alzheimer’s disease, and multiple sclerosis (53). *M. catarrhalis* strains isolated from patients with bronchopulmonary infection were more resistant to complement-mediated killing than strains harbored by healthy carriers (89% vs 41.5%) (54). Furthermore, clinical studies have revealed that the increased prevalence of *M. catarrhalis* infections in the winter is due to the exposure to reduced environmental temperature in the nasopharynx (cold shock response), which increases the expression of virulence factors, including UspA2 (55). Cold-shocked bacteria therefore can bind more C4BP and FH through UspA2 and have an increased resistance to complement. Targeting strains that are able to bind complement inhibitors may thereby select between pathogenic strains and strains part of healthy microbiota.

The development of complement-activating antibodies targeting bacteria represents a promising strategy for antibacterial therapies but is limited by the need to raise specific antibodies against conserved, abundant surface proteins, which are expressed during infection. Such ubiquitous protective epitopes are very difficult to identify, because of the considerable antigenic and phase variability between strains within a species. Making monoclonal antibodies that recognize a wide array of strains and are functional is therefore extremely challenging. The fusion proteins outlined in this study bypass this restriction by behaving as soluble complement inhibitor decoys, which are bound by the majority of clinical isolates. Noteworthy, binding of complement inhibitors is an evasion strategy developed by many different pathogens, from fungi such as *Aspergillus* spp. and *Candida albicans* (56–58), parasites such as *Plasmodium falciparum* (59), and many bacterial species. Among these bacteria, FH6–7/IgG fusion protein successfully enhanced the killing of *H. influenzae* (20), *N. meningitidis* (18), *N. gonorrhoeae* (21), and *Streptococcus pyogenes* (22), while C4BP1–2/IgM strongly enhanced the killing of *N. gonorrhoeae* (19). Therefore, fusion proteins can prove very useful in the treatment of large variety of infections with this study highlighting their effectiveness in enhancing serum killing of *M. catarrhalis*.

## Conclusion

In conclusion, we have shown that fusion proteins combining bacteria-binding sequences of complement inhibitors and Fc parts of immunoglobulins constitute a novel therapeutic approach against the human pathogen *M. catarrhalis*. Targeting such a key immune evasion strategy evolved by bacteria allows us to treat infection without triggering antibiotics resistance, and can be extended to a large variety of human pathogens.

## Data Availability Statement

The raw data supporting the conclusions of this article will be made available by the authors, without undue reservation.

## Ethics Statement

The studies involving human participants were reviewed and approved by the ethical committee in Lund (Permit 2017/582). The patients/participants provided their written informed consent to participate in this study.

## Author Contributions

ML, LC, SB, DE, SR, and AB designed the experiments. ML, LC, SB, and KM conducted the experiments. ML, LC, DE, and AB analyzed the data. KR provided clinical isolates and mutants. ML, LC, and AB wrote the manuscript. All authors contributed to the article and approved the submitted version.

## Conflict of Interest

The authors declare that the research was conducted in the absence of any commercial or financial relationships that could be constructed as a potential conflict of interest. The reviewer EV declared a past co-authorship with one of the authors AB to the handling editor.

## Abbreviations

AF: Alexa Fluor
BHI: brain-heart infusion
BSA: bovine serum albumin
C4BP: C4-binding protein
CCP: complement control protein
CFSE: carboxyfluorescein succinimidyl ester
CFU: colony forming unit
CHO: Chinese hamster ovary
FH: factor H
gMFI: geometric mean fluorescence intensity
GVB: gelatin veronal buffer
Ig: immunoglobulin
MAC: membrane attack complex
NHS: normal human serum
OD: optical density
Usp: ubiquitous surface protein
WT: wild-type.
